# Phenotypic and genomic characterization of antimicrobial resistance in *Escherichia coli* and *Klebsiella spp*. from livestock in Namibia

**DOI:** 10.1371/journal.pone.0359106

**Published:** 2026-10-13

**Authors:** Loide N. Nangolo, Agnetor Kakungu, Sebastian Musundi, Graça K. Kandanda, Samuel Odoyo, Agnes Oclu, Justice Danso, Beverly Egyir, Harris Onywera, George Githinji, Renatus Shilangale, Lamech M. Mwapagha

**Affiliations:** 1 Department of Biology, Chemistry and Physics, Faculty of Health, Natural Resources and Applied Sciences, Namibia University of Science and Technology, Windhoek, Namibia; 2 Epidemiology and Demography Department, KEMRI-Wellcome Trust, Kilifi, Kenya; 3 Bacteriology Department, Noguchi Memorial Institute for Medical Research, University of Ghana, Accra, Ghana; 4 Institute of Infectious Disease and Molecular Medicine, University of Cape Town, Cape Town, South Africa; 5 Division of Medical Microbiology, Department of Pathology, Faculty of Health Sciences, University of Cape Town, Cape Town, South Africa; 6 Department of Biochemistry and Biotechnology, Pwani University, Kilifi, Kenya; 7 Central Veterinary Laboratory, Windhoek, Namibia; Bangladesh Agricultural University, BANGLADESH

## Abstract

**Objectives:**

Antimicrobial resistance (AMR) is a growing concern within the One Health framework. This study characterized AMR in *Escherichia coli* and *Klebsiella* spp. isolated from livestock fecal samples across commercial and communal farms in Namibia.

**Methods:**

About 140 fecal samples were collected from cattle, sheep, and chickens on farms in Namibia. Isolates were identified using the VITEK® 2 system and MALDI-ToF mass spectrometry. Antimicrobial susceptibility testing was performed using the Kirby–Bauer disc diffusion method against nine antibiotics. Genomic DNA was extracted and subjected to whole-genome sequencing (WGS) using Illumina NextSeq 2000 platform.

**Results:**

AMR genes were identified using ResFinder and Abricate, plasmid-associated replicons using plasmid analysis tools, and virulence-associated genes using VirulenceFinder. Phenotypic analyses showed resistance to β-lactams, tetracyclines, aminoglycosides, and fluoroquinolones among the tested isolates. WGS identified diverse resistance determinants and sequence types, including *E. coli* ST101 and ST1431, *K. oxytoca* ST2, and *K. pneumoniae* ST22. Multiple plasmid replicons were detected; however, their presence alone does not establish that individual resistance genes were plasmid-borne or that horizontal gene transfer occurred.

**Conclusions:**

This study provides the first genomic evidence of livestock-associated AMR in Namibia, identifying resistance determinants and multidrug-resistant *E. coli* and *Klebsiella spp.* in the sampled livestock population. The findings provide baseline genomic data for AMR surveillance and support strengthened antimicrobial stewardship and One Health monitoring. The concurrent detection of chromosomal resistance-associated mutations and diverse plasmid replicons illustrates the complexity of resistance mechanisms, although the present short-read data do not establish the direct physical linkage of individual ARGs to plasmids. These findings highlight the need for coordinated surveillance across animal, human, and environmental health sectors in Namibia.

## Introduction

The global rise of antimicrobial resistance (AMR) presents a global public health challenge, driven mainly by the misuse and overuse of antibiotics in healthcare and agriculture. In African countries, the burden of AMR is especially severe due to weak regulations [1], weak surveillance systems, lack of knowledge on proper use of antibiotics [2], use of substandard and counterfeit antibiotics [3] as well as unregulated disposal of antimicrobial waste [4]. Additionally, there is a paucity of reliable data on AMR from African countries to guide evidence-based AMR interventions. While global efforts, such as the Global Antimicrobial Resistance and Use Surveillance System (GLASS), aim to standardize AMR monitoring, their focus has primarily revolved around resistance patterns in humans [5]. Animal reservoirs and environmental sources remain understudied, despite being integral to the One Health framework.

In veterinary practice, antimicrobials are indispensable and are commonly used for treating and controlling diseases and promoting growth. Increasingly, more antibiotics are being used in animal production systems, considering livestock farming is a source of livelihood, especially among most sub-Saharan farmers. This widespread and unregulated use creates stressful conditions for gut microbes, leading to the selection of antibiotic-resistant bacteria, which poses a risk not only to animal health but also to human health through the transmission of resistant bacteria via direct contact, the food chain, or environmental exposure. Namibia was among the first African countries to ban the use of antibiotics as growth promoters and restrict their use in treating bacterial diseases requiring a prescription from a veterinarian, yet over-the-counter access to broad-spectrum antibiotics continues to drive high levels of AMR in livestock [6].

Among bacterial species commonly associated with AMR, *Escherichia coli* and *Klebsiella oxytoca* are of particular interest. Both bacterial species inhabit the gastrointestinal tract of animals and humans but also present as opportunistic pathogens capable of causing disease and becoming zoonotic agents [7,8]. These species also act as reservoirs of resistance genes, acquired through mutations or horizontal gene transfer, which enhances their role in the dissemination of AMR [9]. There remains limited genomic evidence on AMR in Namibian livestock, thereby creating a critical knowledge gap.

This study aimed to address this gap by providing the first whole-genome sequencing (WGS)-based characterization of livestock-associated AMR in the country

## Materials and methods

### Inclusivity in global research

Additional information regarding the ethical, cultural, and scientific considerations specific to inclusivity in global research is included in the Supporting Information (SI Checklist).

### Ethical considerations, permits and authorship

Permissions and/or consent for the study to take place as well as ethical approval for sample collection and animal handling was granted by the Namibia University of Science and Technology (NUST). Written informed consent was obtained from all livestock farm owners prior to sampling and collection procedures followed applicable animal welfare guidance, adhering to OIE guidelines on animal welfare.

### Study design and sample collection

The study involved random sampling of animals from three commercial farms (Ka, Ne) around Khomas region, one in Otjozondjupa region (farm Om), and homestead/communal farms for the controls from Omusati region (Outapi constituency). Farm Ka reared chickens only, while Farm Ne and Farm Om reared cattle, goats, sheep, and chickens. For the control group, samples were collected from small-scale homestead/communal farms, where cattle, chicken, goats, and sheep are raised alongside subsistence farming. The samples were collected in November 2022.

Fresh fecal samples were collected directly from the rectum of cattle and swabs from sheep, goats, and the cloaca of chicken. A total of 140 samples from 118 animals were aseptically collected from the different farms and immediately transferred into sterile Falcon tubes (faecal samples) or zip-lock bags (swab samples). All samples were transported to the laboratory under cooled conditions and stored at 2–8 °C upon arrival until further analysis (Table 1).

**Table 1:** Distribution of animal faecal samples collected from different farms in Namibia.

Farm/ Source	Cattle	Sheep	Goats	Chickens	Total Samples
OM Farm	16	13	16	5	**50**
Ne Farm	9	9	10	9	**46**
Ka Farm	0	0	0	17	**17**
Homestead/ Communal	5	3	6	0	**27**
Total number of animals	**30**	**25**	**32**	**31**	

### Bacteria Isolation

Bacterial isolation followed Irshad et al. (2016), with minor modifications. Briefly, 1 g of each fecal and swab sample was aseptically transferred into 3 ml of buffered peptone water (BPW) for pre-enrichment and bacterial resuscitation. The inoculated samples were allowed to stand at room temperature (approximately 25°C) for one hour to facilitate the recovery of stressed or injured bacterial cells. The samples were incubated at 37°C for 24 hours to allow sufficient bacterial growth before further processing. Following incubation, 10 μl of each sample was streaked onto MacConkey agar (S1 Fig). Samples were then incubated at 37°C for 24 hours. Suspected isolates with pink to red colonies were again sub-cultured on brilliant green agar and incubated at 37°C for 24 hours (S1 Fig). The isolates were stored in 50% glycerol stocks at −80°C for further molecular and antimicrobial susceptibility analyses.

### Identification and confirmation of the bacterial isolates by VITEK automated machine

The presumptive *E. coli* and *K. oxytoca* isolates were identified and confirmed using the VITEK® 2 Compact system bioMérieux. The bacterial suspensions were prepared in 0.45% saline to a turbidity equivalent to a 0.5 McFarland standard. The Gram-negative identification VITEK cards were loaded in the suspensions to identify the bacteria, according to the manufacturer’s instructions. Identification was based on biochemical profiles that were automatically interpreted using the VITEK database. Results were reported with confidence levels provided by the system.

### Bacterial identification via MALDI-TOF mass spectroscopy (MS)

Briefly, a thin film of bacteria isolate was prepared on the MALDI target plate with a fresh overnight bacterial culture. This was done with a sterile 1 µL inoculation loop. The sample spot was overlaid with 1 µL 70% formic acid and allowed to dry at room temperature. A total of 1 µL drop of Bruker HCCA solution was also added and allowed to air dry at room temperature. The MALDI target plate was loaded into the MALDI Biotyper, and MALDI-TOF-MS measurements were taken.

### Phenotypic antimicrobial susceptibility testing (AST)

AST was done following the Kirby-Bauer Disk Diffusion method, in accordance with the Clinical and Laboratory Standards Institute (CLSI) and National Committee for Clinical Laboratory Standards (NCCLS) guidelines for interpretation (S1 Table). The resistance of the isolates was examined against nine antibiotics, namely: Tetracycline (Tet, 30 µg), Ampicillin (Amp, 10 µg), Kanamycin (Kan, 30 µg), Ceftazidime (Cef, 30 µg), Erythromycin (Ery, 15 µg), Ciprofloxacin (Cip, 10 µg), Amoxicillin/clavulanic acid (AMX, 30 µg), Vancomycin (Van, 30 µg), and Cefuroxime (CXM, 30 µg),. From a pure bacterial culture (*E. coli* and *K. oxytoca*), freshly incubated, a loop full of colonies was obtained and transferred to 5 ml of Trypticase soy broth and mixed using a vortex to a concentration of approximately 0.5 McFarland. The plates were then inoculated, whereby 200 µL bacterial suspension was added to the Mueller-Hinton media plate and spread all over the plate by rotating the plate at 60° rotation to ensure an even distribution of the inoculum. The surface of the medium was allowed to dry for 3–5 minutes, but not longer than 15 minutes, to allow for absorption of excess moisture. Using sterile forceps, antibiotic discs were placed on the surface of the inoculated plate and immediately pressed down lightly to ensure complete contact between the disc and the agar surface. The discs were positioned such that the minimum centre-centre distance was 24 mm and no closer than 10–15 mm from the edge of the petri dish. The plates were then incubated in an inverted position at 37°C, and the zone of inhibition was observed and measured after 16–18 hours. The results were recorded as either resistant, intermediate, or susceptible based on the CLSI guidelines (Standards & Testing, 2020).

### DNA extraction and whole genome sequencing

DNA extraction was done from pure colonies of overnight cultures using the QIAamp DNA Mini Kit (Qiagen, Germany). DNA concentration was measured with the Qubit® dsDNA High Sensitivity assay and fluorometer (version 4.0) (Life Technologies, Carlsbad, CA, USA) to ensure a minimum of 500ng was obtained for sequencing. Library preparation followed the Illumina DNA Library Preparation Reference Guide (Illumina Inc., San Diego, CA, USA) using the Illumina DNA Library Preparation Kit. This process included DNA fragmentation and tagmentation, addition of index sequences, amplification of indexed fragments, and fragment size selection. Library quality was assessed with the 2100 Bioanalyzer system (Agilent), and concentrations were determined using qPCR (Kapa SYBR Fast qPCR kit). Individual libraries were normalized and pooled at a final concentration of 2nM, using 2 × 150 bp paired-end chemistry, before sequencing on the NextSeq 2000 platform (Illumina Inc., San Diego, CA, USA).

### Bioinformatics analysis

Raw read quality was assessed using FastQC v0.21.1 [10]. Samples with fewer than 1,000 reads were excluded from further analysis. Adapter trimming and removal of low-quality bases (Phred score <20) were performed using Trimmomatic v0.39 [11]. Taxonomic classification of individual samples was conducted using Centrifuge v1.0.4 (https://github.com/DaehwanKimLab/centrifuge) against prokaryotic, archaea, and virus databases. The resulting reads were then assembled using SPAdes v3.13.1 [12], and the quality of the assembled genomes was assessed using Quast v5.3.0 [13]. Assemblies with fewer than 300 contigs and an N50 > 25,000 were used for downstream analysis.

For *E. coli* isolates, Multi Locus Sequence Typing (MLST) v2.22.0 and ClermonTyping (http://clermontyping.iame-research.center/) were used for sequence type (ST) assignment and phylogenetic classification, respectively. For *Klebsiella* spp., Kleborate v3 [14] was utilized for typing K and O loci in and identifying the associated virulence factors. Acquired and chromosomal ARGs were detected with ResFinder v4.6.0 (http://genepi.food.dtu.dk/resfinder) and virulence genes with VirulenceFinder v2.0 (https://cge.food.dtu.dk/services/VirulenceFinder/). Plasmids were reconstructed for all samples using the MOB-recon module from MOB-suite v3.1.9 [14]. The reconstructed plasmid replicons were subsequently analyzed for ARGs using ResFinder.

Core genome alignment for *E. coli* was generated using Snippy v4.3.6 [15], with NC_016845.1 as the reference. Alignments were curated by removing ambiguous bases and recombinant regions with Gubbins v3.4 [16]. Single-nucleotide polymorphisms (SNPs) were extracted using Snp-sites v2.5.1 [17] and maximum likelihood phylogenies were inferred IQ-TREE v2.4.0 [18] under the general time reversible (GTR) model with 1,000 bootstrap replicates. Resulting trees were annotated and visualized in R v4.4.3 [19].

For *Klebsiella* species (*K. pneumoniae* and *K. oxytoca*), genome annotation was performed using Prokka v1.14.6 [20] (parameters: kingdom Bacteria, --genus *Klebsiella*). The generated general feature format (GFF3) v3 served as input in Roary v3.13.0 [21] for generating the pangenome. The core genome alignment was retrieved and used as input for constructing a maximum likelihood trees in IQ-TREE v2.4.0 [22], with the same parameters applied to the *E. coli* phylogeny.

## Results

### Bacterial isolates and antimicrobial susceptibility pattern

To determine the prevalence of enteric bacteria in livestock and their resistance to commonly used antibiotics, we cultured fecal samples, identified isolates with VITEK and MALDI-ToF, and performed AST against nine clinically relevant antibiotics. From the 140 fecal samples collected across 27 farms and homesteads in Namibia*, Escherichia coli* and *Klebsiella oxytoca* were the most frequently isolated species, with 25 and 32 confirmed isolates, respectively. Phenotypic assays revealed multidrug resistance (MDR) (S2 Fig). *E. coli* exhibited resistance to multiple antibiotics, including ampicillin, tetracycline, and cefuroxime, as indicated by the absence of inhibition zones around the discs. Similarly, *K. oxytoca* was resistant to amoxicillin/clavulanic acid and vancomycin but remained largely susceptible to ciprofloxacin, evident from the distinct inhibition zone around the ciprofloxacin disc. Although vancomycin and erythromycin are included in the AST interpretation, they were only included for screening purposes but not for MDR as they intrinsically inactive against enterobacterales.

After positive confirmatory test, the antibiotic resistance patterns of *E. coli* (n = 13), *K. oxytoca* (n = 9), and *Klebsiella spp*. (n = 5) to the nine clinically relevant antibiotics were examined and reported (Fig 1). *E. coli* showed complete resistance (100%) to seven drugs (Amp, CXM, Van, Ery, Kan, Cef, and AMX), and partial resistance to Cip (approximately 25%). *K. oxytoca* isolates exhibited complete resistance (100%) resistance to six drugs (Amp, CXM, Van, Ery, Kan, and AMX) and partial resistance to Cef (25%) and Tet (25%). *Klebsiella* spp. near-complete (100%) resistance across all the drugs, with only Tet (80%) and Cef (approximately 60%) showing varied activity.

**Fig 1:**
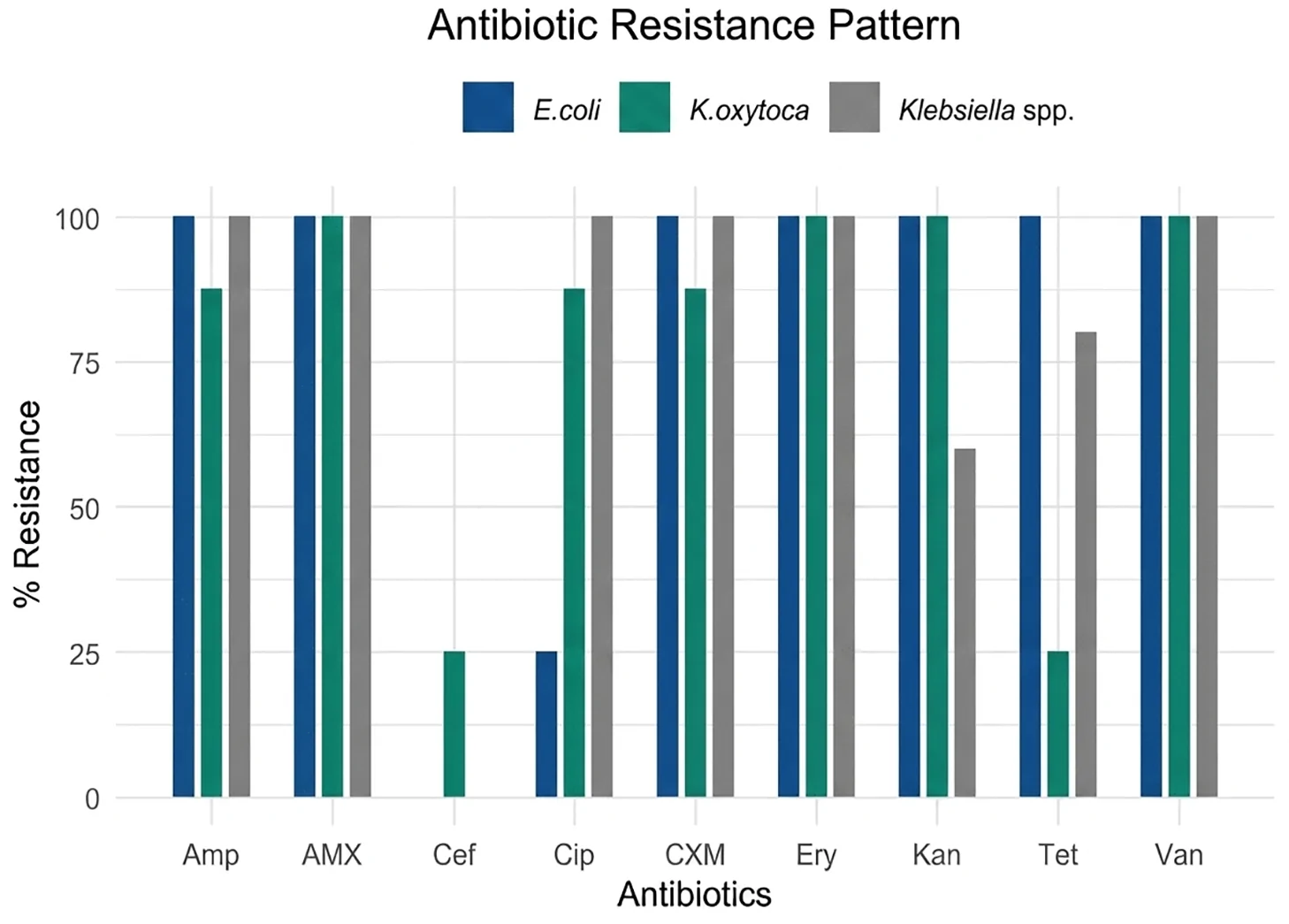
Antibiotic resistance patterns of *E. coli*, *K. oxytoca*, and *Klebsiella* spp. against common antibiotics, namely tetracycline (Tet), ampicillin (Amp), cefuroxime (CXM), ciprofloxacin (Cip), vancomycin (Van), erythromycin (Ery), kanamycin (Kan), ceftazidime (Cef), and amoxicillin-clavulanic acid (AMX). Vancomycin and erythromycin are shown as screening observations but were excluded from MDR classification.

### Genomic characteristics

To characterize the genomic composition of the recovered isolates, we performed metagenomic sequencing, taxonomic classification, and genome assembly quality assessment. Taxonomic classification revealed five types of microorganisms present in the samples. *E. coli* was the highest in number (n = 12), followed by *K. oxytoca* (n = 7), *Klebsiella* spp. (*K. michiganensis* = 6; *K. pneumoniae* = 4), and *Raoultella ornithinolytica* (n = 1). Four samples were dropped due to the low number of reads (<1,000) (S2 Table). After excluding the four low-coverage samples, 26 genomes remained *E. coli* (n = 12)*, K. oxytoca* (n = 7), *Klebsiella* spp. (*K. michiganensis* = 6; *K. pneumoniae* = 1) (S2 Table).

The genomic characteristics of the samples, as analyzed using QUAST, are presented in S2 Table. The assembled contig sizes (largest contig per sample) ranged from 0.02 to 0.7 Mb, with GC content values between 50.5% and 55.8%. Genomes with more than 300 contigs and an N50 below 25,000 bp were excluded from subsequent phylogenetic and antimicrobial resistance gene analyses. (Quast_on_data_1428,_data_1424,_and_others__HTML_report_html.html)

### High-Risk Multidrug-Resistant Clones Identified in *E. coli* Isolates in Cattle and Chicken

To explore the genetic diversity and resistance determinants, we applied MLST typing, phylogroup classification, and resistome/virulome profiling. The MLST analysis in *E. coli* from livestock feces across farms and homesteads revealed considerable diversity, identifying eight sequence types (STs): ST101, ST1431, ST154, ST1727, ST181, ST20, ST2005, and ST2280. In addition, phylogroup B1 was most common in the samples (87.5%; n = 7/8) and A1 (12.5%; n = 1/8). A high-risk clone, ST101, was detected in the *E. coli* sample isolated from homestead cattle samples. It carried multiple ARGs conferring resistance to: aminoglycosides (*aph(6)-Id*, *aph(3’‘)*-*Ib, aph(3’)-Ia*), and tetracyclines (*tet(A)*), beta (β-lactams, trimethoprim, plus multiple plasmid replicons (Fig 2A, Fig 3). ST1431 was also identified in chicken waste samples, a clone previously isolated from livestock samples, and had ARGs such as tet*(A)*, *sul1*, and *dfrA5* alongside a plasmid replicon; rep_cluster_2335 (Fig 2A). Additionally, it carried chromosomal-encoded mutations in DNA gyrase (*gyrA*) and topoisomerase IV (*parC* and *parE*), linked to quinolone resistance.

**Fig 2:**
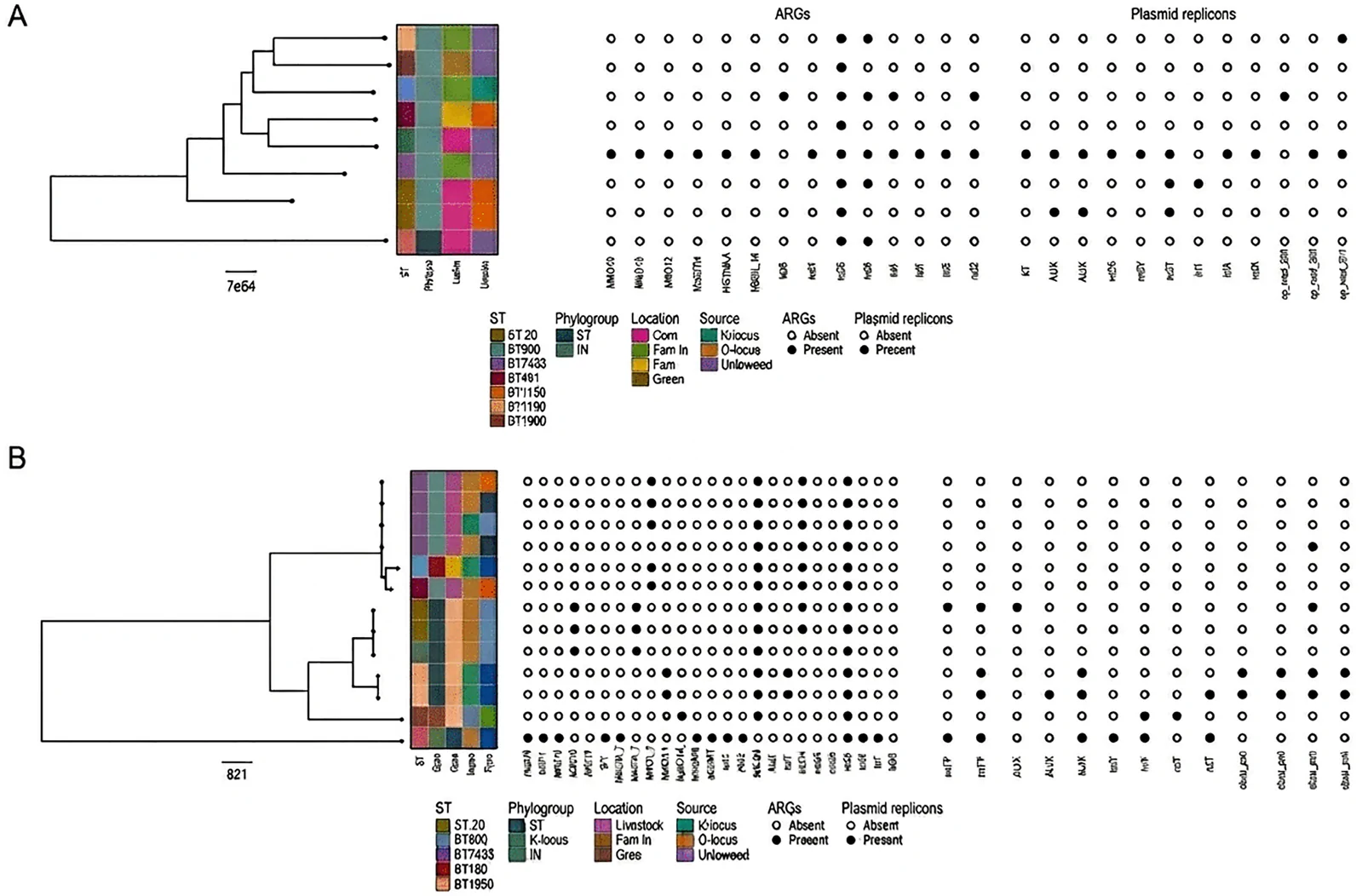
*E. coli* and *Klebsiella spp.* core-genome maximum likelihood tree annotated using ARGs and plasmid replicons. A) E. coli isolates annotated based on their sequence types, phylogroups, sample location, livestock source, sequence types, presence and absence of ARGs, and plasmid replicons. B) Klebsiella spp. annotated based on sequence types, K-locus, O-locus, sample location, type of livestock, and presence or absence of ARGs, and plasmid replicons.

**Fig 3:**
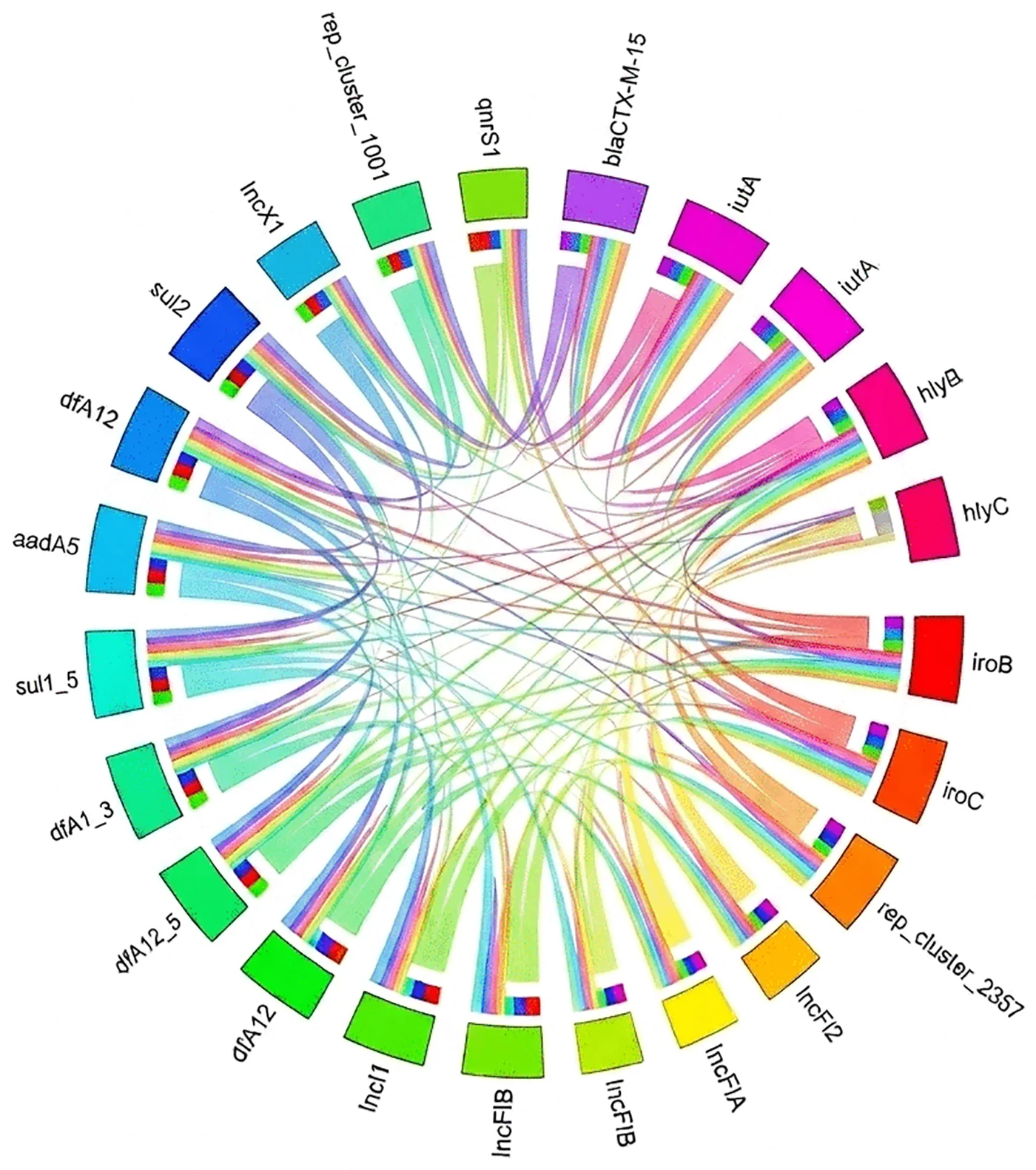
Descriptive co-occurrence network showing associations among AMR genes, plasmid replicon types, and virulence factors detected in *E. coli* isolates from livestock in Namibia. Nodes represent individual genetic features and connecting edges represent the relationships included in the network. Because of the small number of genomes, the network is presented as an exploratory descriptive visualization and should not be interpreted as evidence of statistically significant associations, physical ARG–plasmid linkage, or horizontal gene transfer.

Further analysis revealed that all *E. coli* isolates contained multiple multidrug efflux pumps (*acrD, acrE, acrS, tolC, emrA, mdtB–mdtO,* and *msbA*), β-lactamase genes (*ampC* and *ampH*) as well as cell wall lipid modification genes (*bacA* and *eptA*), and drug inactivating enzymes (*mphB* (S3 Table). Additionally, the most commonly identified virulence factors, present in at least 9/11 isolates, included those mediating adhesion and biofilm formation (*csgB*, *csgD*, *csgF*, *fimA–I*, *fdeC*, *ompA*, *ecpA–R*), iron acquisition (*entA–F*, *entS*, *fes*, *fepA–G*, and *fepB–D*), outer membrane and serum resistance (*ompA, fdeC*), and secretion proteins (*gspC–M (T2SS)*, *espL1*, *espX1/X4/X5*, and *espR1 (T3SS)*) (S4 Table).

### Detection of MDR *K. pneumoniae* ST22 and Clonal Patterns in the *K. oxytoca* Complex

To investigate clonal relatedness and resistance determinants within *Klebsiella* spp., we performed MLST, capsular (K) locus and O-antigen typing, and resistome analysis. This identified MDR *K. pneumoniae* ST22 and distinct clonal patterns within the *K. oxytoca* complex. All *K. oxytoca* isolates harboured intrinsic ARGs (*bla*_*OXY*_ and *fosA* genes), which confer resistance to β-lactams and fosfomycin. ST2, a β-lactam-resistant clonal complex (CC), was the most common ST in *K. oxytoca,* present in 4 out of 6 isolates (66.7%). These ST2 isolates also shared the same K locus KL148 and O-antigen (O-locus) 0L0, despite being isolated from different animal hosts—cattle, chicken, and goats—in two other farms (Fig 2B). *K. michiganensis*, which is part of the *K. oxytoca* complex, showed diversity in ST types (ST11, ST29), but was mainly characterized by the capsular locus KLO7 and O-antigen 01/02v1 (Fig 2B). Of the 3 out of 6 *K. michiganensis* isolates (50.0%) from cattle from the same farm carried *aph(3”)-lb* and bla_CTX-M-55_, which confer resistance to aminoglycosides and β-lactams, respectively, while the remaining isolates (50.0%) from sheep from different farms showed the same ARG profile (Fig 2B). One *K. pneumoniae* isolate from chicken, belonging to the high-risk clone ST22 with capsular locus KL9 and O-antigen 01/02v2, was detected in one of the farms. This isolate exhibited multidrug resistance, carrying genes conferring resistance to aminoglycosides (*aac(6”)-lb, aadA-16, aph(3”lb), aph(6”ld)*), β-lactams (bla_CTX-M-55,_ blaSHV-_187,_ bla_TEM-1_), chloramphenicol (*catA2*), trimethoprim (*dfrA27*), sulfonamides (*sul1*), and tetracycline (*tet(A)*) (Fig 2B, Fig 4). Several genes related to stress and heavy metals were identified in the samples. Heavy metal resistance genes (*czcA*, *zraP*, *arsR2*, *copD*, *arsD_1*, *cnrA*) conferring resistance to copper, zinc, and arsenite were identified across isolates.

**Fig 4:**
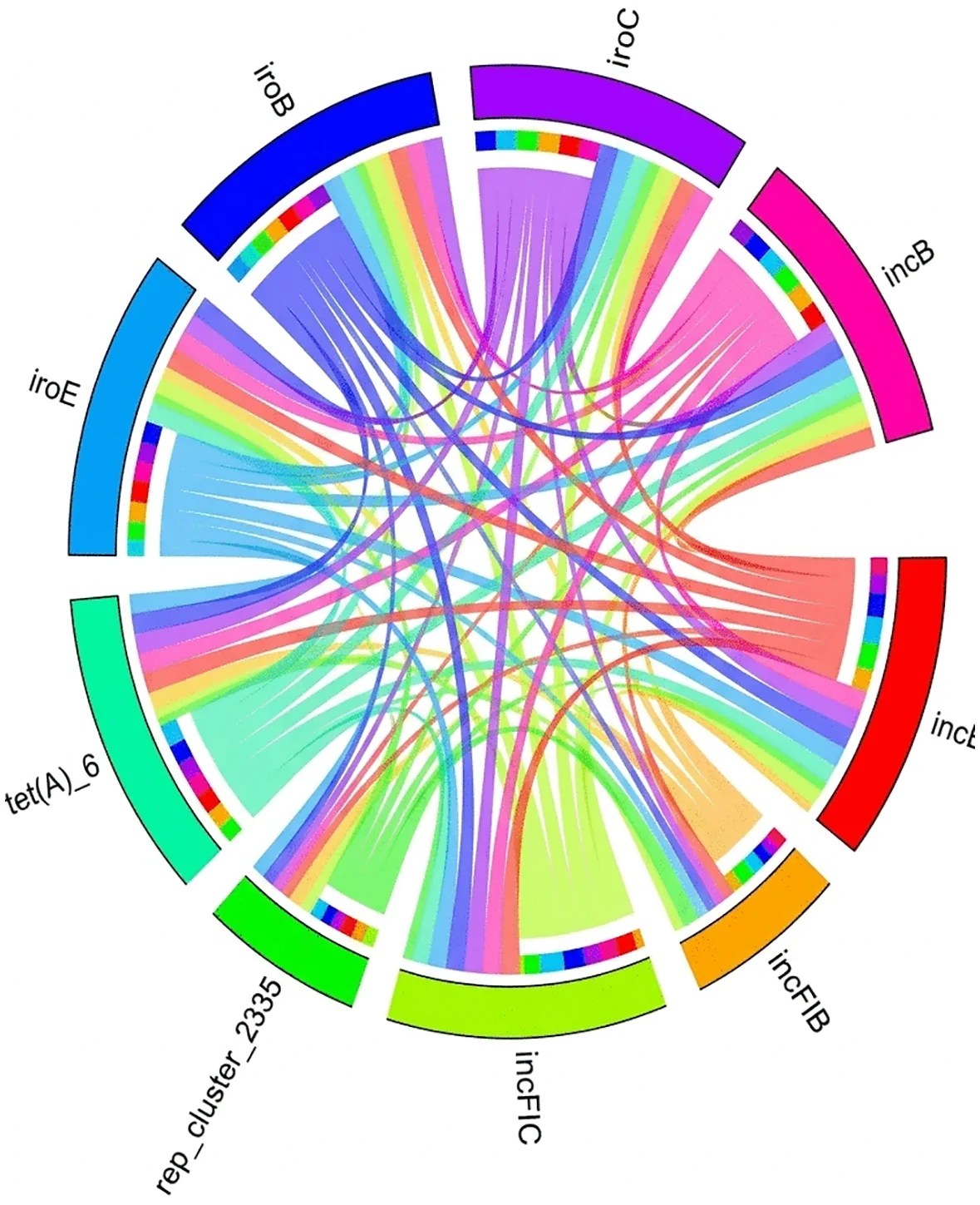
Descriptive co-occurrence network showing relationships among AMR genes, plasmid replicon types, and virulence-associated factors identified in *Klebsiella spp*. isolates from livestock in Namibia. Each node represents a genetic feature and edges represent relationships included in the exploratory network. Given the small number of genomes, the figure should be interpreted descriptively rather than as evidence of statistically significant associations, physical ARG–plasmid linkage, or horizontal gene transfer.

## Discussion

Our study provides novel genomic and phenotypic insights into antimicrobial resistance (AMR) among *Escherichia coli* and *Klebsiella oxytoca* isolated from livestock in Namibia an underrepresented region in global AMR surveillance. Both species are recognized reservoirs of antimicrobial resistance genes (ARGs) and opportunistic pathogens capable of transmitting resistance determinants across human, animal, and environmental interfaces within the One Health framework. For instance, *E. coli*, a common commensal inhabitant of the gastrointestinal tract (GIT) of animals, can readily acquire and disseminate ARGs and virulence factors [23]. Moreover, *E. coli* populations within the GIT can undergo dynamic shifts, with certain strains persisting or proliferating in response to ecological and selective pressures.

Conversely, *K. oxytoca* is a clinically relevant opportunistic pathogen capable of causing various infections, including urinary tract and respiratory infections. Despite their significance, there is limited genomic data on the distribution of ARGs and the clonal diversity of these organisms, especially in livestock from low- and middle-income countries, including Namibia. To address this gap, we examined ARG profiles of *E. coli* and *Klebsiella* spp. from fecal samples collected across multiple livestock farms in Namibia.

The phenotypic assays confirmed high levels of MDR, particularly to β-lactams and tetracyclines, reflecting the potential use of antibiotics within Namibian settings as well as their potential dissemination through plasmids. The observed phenotypic resistance was comparable for both *E. coli* and *K. oxytoca* to the commonly used antibiotics. This is in line with previous studies in China, which have shown *E. coli* resistance against commonly used antibiotics, including ampicillin, tetracycline, and ciprofloxacin [24]. The observed resistance presents a health concern, with most of the tested antibiotics being listed in the World Health Organization (WHO) priority list. Interestingly, *K. oxytoca* isolates displayed consistent resistance across multiple antibiotic classes but retained susceptibility to ciprofloxacin, suggesting that the fluoroquinolones may still provide therapeutic value in this setting. However, the observed resistance to commonly used veterinary and clinical antibiotics, including the ones used in this study, attests to a shrinking therapeutic arsenal and the urgent need of antimicrobial stewardship.

Genomic analyses revealed the presence of high-risk MDR clones, including *E. coli* ST101, ST1431, which has been linked to extra intestinal infections and has been previously associated with a major poultry outbreak in Denmark. Its presence in settings with low-antibiotic use on homesteads is particularly concerning, as it suggests the persistence of resistant clones in the environment, transmission through contaminated water or human-animal contact, and co-selection via horizontal gene transfer of resistance genes, as indicated by the presence of multiple plasmid replicons [25].

The phenotypic assays demonstrated resistance to multiple antimicrobial classes, particularly β-lactams and tetracyclines, among the AST-tested isolates. These findings are consistent with antimicrobial selection pressure being a possible contributor, but antimicrobial-use data were not collected systematically at farm level and therefore causal relationships cannot be established. The observed resistance is broadly consistent with previous reports of resistance to commonly used antibiotics in farm-associated *E. coli* [24]. The findings reinforce the need for antimicrobial stewardship and continued surveillance in livestock production.

Genomic analyses identified resistance-associated sequence types, including *E. coli* ST101 and ST1431. ST1431 has previously been reported in livestock-associated contexts [25]. Their detection in the present study demonstrates that these lineages occur in the sampled Namibian livestock population; however, the small number of isolates and absence of comparative epidemiological data do not permit conclusions about transmission, persistence, outbreak relevance, or human-animal spread. The presence of multiple plasmid replicons suggests that mobile genetic elements may be relevant to the observed resistome, but short-read assemblies do not establish physical linkage between specific ARGs and individual plasmids.

In our study, *K. oxytoca* isolates belonged to ST2, a lineage within clonal complex 2 (CC2) that has been described in AMR-associated and healthcare-associated contexts [26]. The repeated observation of ST2 and similar K- and O-locus profiles across sampled hosts is notable, but the small sample size does not establish adaptation, transmission, or persistence across farms. Similar resistance profiles among *K. michiganensis* isolates from cattle and sheep were observed, but systematic antimicrobial-use data were not available to determine whether these patterns were associated with livestock-specific antibiotic practices. The co-occurrence of a *K. pneumoniae* ST22 isolate with *K. michiganensis* in the same farm is likewise an observation requiring further investigation rather than evidence of transmission or environmental co-selection.

While our study has added knowledge to the regional gap in genomic and phenotypic data on *E. coli* and *Klebsiella* spp. from livestock in Namibia and, more broadly, southern Africa, some key limitations must be acknowledged. First, the relatively small sample size and focus on three farms restrict generalizability across Namibia. Second, reliance on culturable isolates may underestimate the diversity of resistance present in uncultured microbial communities. Third, the absence of systematic farm-level antibiotic usage data limits the ability to link resistance phenotypes directly to antimicrobial exposure. Finally, the cross-sectional design prevents assessment of temporal trends in AMR evolution or persistence. Thus, larger, longitudinal studies across diverse farms, including uncultured microbial communities and systematic farm-level antibiotic usage data, are needed to link resistance phenotypes to antimicrobial exposure and track temporal trends in AMR. Despite these limitations, our results indicate that Namibian farming practices contribute to the emergence and maintenance of multidrug-resistant bacteria and lay the foundation for future genomic surveillance and understanding of AMR dynamics in Namibia.

## Conclusion

This study characterized phenotypic and genotypic AMR among *E. coli* and *Klebsiella spp.* isolated from livestock in Namibia and provides the first genomic evidence of livestock-associated AMR in the sampled setting. The study identified multiple resistance determinants and sequence types, including *E. coli* ST101 and *K. pneumoniae* ST22, as well as diverse plasmid replicons. These findings provide a baseline for future AMR genomic surveillance in Namibia. Because farm-level antimicrobial-use data were not systematically collected and the study was cross-sectional with a relatively small genomic dataset, the observed resistance patterns cannot be attributed to specific farming practices or interpreted as evidence of transmission. Continued surveillance, responsible antimicrobial use, and integrated One Health monitoring are warranted, together with larger longitudinal studies that incorporate antimicrobial-use data and improved characterization of mobile resistance elements.

## Supporting information

S1 Fig Bacteria cultures on selective media. A, B, C, and D show red to pink colonies on MacConkey agar, indicating the presence of *E. coli*. E, F, and G show mucoid pink colonies on MacConkey agar, indicating the presence of *K. oxytoca*. H shows green metallic sheen colonies on Eosin Methylene Blue agar, indicating the presence of *E. coli*. The red-to-pink colonies observed on MacConkey agar indicate lactose fermentation, characteristic of *E. coli*. MacConkey agar contains bile salts and crystal violet, which inhibit Gram-positive bacteria while differentiating lactose fermenters (pink colonies) from non-fermenters (colorless colonies). The green metallic sheen colonies observed on EMB agar further confirm the presence of *E. coli*. EMB agar differentiates strong lactose fermenters, such as *E. coli*, which produce acid that precipitates dyes in the medium, forming a distinctive metallic sheen.
(DOCX)

S2 Fig The clear zones around antibiotic discs indicate susceptibility, while the absence of inhibition zones confirms resistance. (A) showing the resistance of *E. coli* against ampicillin, tetracycline, and cefuroxime. (B) showing resistance of *K. oxytoca* against amoxicillin/clavulanic acid and vancomycin as well as susceptibility against ciprofloxacin. The Kirby-Bauer disc diffusion results showed a high level of resistance to multiple antibiotic classes, particularly β-lactams (ampicillin, amoxicillin-clavulanic acid, and cefuroxime), tetracyclines, and aminoglycosides. *E. coli* exhibited resistance to ampicillin (Amp), tetracycline (Tet), and cefuroxime (CXM), while *K. oxytoca* showed resistance to amoxicillin-clavulanic acid (AMX) and vancomycin (Van). However, *K. oxytoca* isolates demonstrated susceptibility to ciprofloxacin (Cip), suggesting the potential effectiveness of fluoroquinolones against this species.
(DOCX)

S1 Table Standard interpretative categories and zone diameter breakpoints for Enterobacterales as per Clinical & Laboratory Standards Institute guidelines. (DOCX)

S2 Table Quality assessment of the generated assemblies. (DOCX)

S3 Table CARD output indicating some of the intrinsic chromosomal genes detected which contribute to resistance using multiple pathways. (DOCX)

S4 Table Virulence genes derived from *E. coli* isolates using the VFDB database. (DOCX)
