# Supplementary material for: Phenotypic and genomic characterization of antimicrobial resistance in Escherichia coli and Klebsiella spp. from livestock in Namibia
Source: PLoS One. 2026 Oct 13;21(10):e0359106. doi: 10.1371/journal.pone.0359106 (PMC13660907; doi:10.1371/journal.pone.0359106)
Supplement: Supplementary file 1 [file pone.0359106.s001.docx]

**S1.** Bacteria cultures on selective media. A, B, C, and D show red to pink colonies on MacConkey agar, indicating the presence of *E. coli.* E, F, and G show mucoid pink colonies on MacConkey agar, indicating the presence of *K. oxytoca.* H shows green metallic sheen colonies on Eosin Methylene Blue agar, indicating the presence of *E. coli.* The red-to-pink colonies observed on MacConkey agar indicate lactose fermentation, characteristic of *E. coli*. MacConkey agar contains bile salts and crystal violet, which inhibit Gram-positive bacteria while differentiating lactose fermenters (pink colonies) from

non-fermenters (colorless colonies) (Centers for Disease Control & Prevention, 2023). The green metallic sheen colonies observed on EMB agar further confirm the presence of *E. coli*. EMB agar differentiates strong lactose fermenters, such as *E. coli*, which produce acid that precipitates dyes in the medium, forming a distinctive metallic sheen (Brooks et al., 2023).
