# Supplementary material for: Phenotypic and genomic characterization of antimicrobial resistance in Escherichia coli and Klebsiella spp. from livestock in Namibia
Source: PLoS One. 2026 Oct 13;21(10):e0359106. doi: 10.1371/journal.pone.0359106 (PMC13660907; doi:10.1371/journal.pone.0359106)
Supplement: Supplementary file 2 [file pone.0359106.s002.docx]

**Supporting information**

**S2.** The clear zones around antibiotic discs indicate susceptibility, while the absence of inhibition zones confirms resistance. (A) showing the resistance of *E. coli* against ampicillin, tetracycline, and cefuroxime. (B) showing resistance of *K. oxytoca* against amoxicillin/clavulanic acid and vancomycin as well as susceptibility against ciprofloxacin. The Kirby-Bauer disc diffusion results showed a high level of resistance to multiple antibiotic classes, particularly β-lactams (ampicillin, amoxicillin-clavulanic acid, and cefuroxime), tetracyclines, and aminoglycosides. *E. coli* exhibited resistance to ampicillin (Amp), tetracycline (Tet), and cefuroxime (CXM), while *K. oxytoca* showed resistance to amoxicillin-clavulanic acid (AMX) and vancomycin (Van). However, *K. oxytoca* isolates demonstrated susceptibility to ciprofloxacin (Cip), suggesting the potential effectiveness of fluoroquinolones against this species**.**
