# Supplementary material for: Phenotypic and genomic characterization of antimicrobial resistance in Escherichia coli and Klebsiella spp. from livestock in Namibia
Source: PLoS One. 2026 Oct 13;21(10):e0359106. doi: 10.1371/journal.pone.0359106 (PMC13660907; doi:10.1371/journal.pone.0359106)
Supplement: Supplementary file 3 [file pone.0359106.s003.docx]

**Supplementary Table 1.** Standard interpretative categories and zone diameter breakpoints for *Enterobacterales* as per Clinical & Laboratory Standards Institute guidelines.

| Antibiotic | **Abbreviation** | **Antibiotic sensitivity rating (mm)** | | |
| --- | --- | --- | --- | --- |
|  |  | **Susceptible (S)** | **Intermediate (I)** | **Resistant (R)** |
| Ampicillin | AMP | ≥17 | 14–16 | ≤13 |
| Ciprofloxacin | CIP | ≥26 | 22–25 | ≤21 |
| Tetracyclines | TET | ≥15 | 12–14 | ≤11 |
| Cefuroxime | CXM | ≥18 | 15–17 | ≤14 |
| Ceftazidime | CEF | ≥21 | 18–20 | ≤17 |
| Amoxicillin –  clavulanic acid | AMC | ≥18 | 14–17 | ≤13 |
| Kanamycin | KAN | ≥18 | 14–17 | ≤13 |
| Erythromycin | ERY | - | - | - |
| Vancomycin | VAN | - | - | - |

NB: (-); The diameter zone breakpoint values for these antibiotics are not reported in the guidelines for that bacteria family.
