# Supplementary material for: Phenotypic and genomic characterization of antimicrobial resistance in Escherichia coli and Klebsiella spp. from livestock in Namibia
Source: PLoS One. 2026 Oct 13;21(10):e0359106. doi: 10.1371/journal.pone.0359106 (PMC13660907; doi:10.1371/journal.pone.0359106)
Supplement: Supplementary file 4 [file pone.0359106.s004.docx]

**Supplementary Table 2.** Quality assessment of the generated assemblies.

| **Sample Names** | **Number**  **of contigs** | **Largest**  **contig** | **Total**  **length** | **GC-**  **content**  **(%)** | **N50** |
| --- | --- | --- | --- | --- | --- |
| 10-Ecoli_S19_L001 | 66 | 394989 | 5713365 | 55.21 | 216628 |
| 38-Koxy_S31_L001 | 49 | 636085 | 5713835 | 55.22 | 358526 |
| 39-KlebOxytoca_S1_L001 | 133 | 374195 | 5104698 | 50.75 | 137519 |
| 113-Ecoli_S2_L001 | 61 | 672033 | 4733603 | 50.54 | 209889 |
| 44-Koxy_S21_L001 | 43 | 476456 | 5940064 | 55.83 | 232216 |
| 45-Koxy_S32_L001 | 220 | 202261 | 5249770 | 50.56 | 71076 |
| 47-Koxy_S29_L001 | 528 | 65518 | 5914185 | 55.83 | 18796 |
| 48-Kpneuomo_S7_L001 | 53 | 534871 | 5711817 | 55.22 | 260965 |
| 49-Koxy_S28_L001 | 66 | 394989 | 5713365 | 55.21 | 216628 |
| 55-Ecoli_S13_L001 | 1529 | 45310 | 4818234 | 50.6 | 4601 |
| 60-Koxy_S11_L001 | 49 | 636085 | 5713835 | 55.22 | 358526 |
| 61-Koxy_S30_L001 | 3213 | 9074 | 4795643 | 54.93 | 1839 |
| 64-Koxy_S12_L001 | 140 | 494193 | 6057371 | 55.6 | 99422 |
| 70-Koxy_S22_L001 | 133 | 309696 | 5970287 | 55.63 | 117291 |
| 71-Koxy_S23_L001 | 2826 | 12532 | 5168113 | 54.97 | 2318 |
| 75-Ecoli_S15_L001 | 42 | 719913 | 4981391 | 50.57 | 415633 |
| 77-Koxy_S24_L001 | 59 | 545942 | 570917 | 55.22 | 285254 |
| 78-Ecoli_S16_L001 | 187 | 217393 | 5050654 | 50.51 | 72929 |
| 91-Ecoli_S14_L001 | 78 | 54190 | 4616160 | 50.82 | 236052 |

| 105-Ecoli_S4_L001 | 46 | 641327 | 4654104 | 50.86 | 267173 |
| --- | --- | --- | --- | --- | --- |
| 106-Ecoli_S3_L001 | 123 | 412887 | 5046264 | 50.54 | 149350 |
| 109-Ecoli_S5_L001 | 104 | 293390 | 4654402 | 50.93 | 132421 |
| 112-Ecoli_S6_L001 | 50 | 845586 | 4603974 | 50.88 | 335428 |
| 122-Kpneumo_S9_L001 | 139 | 358715 | 4931054 | 50.64 | 97051 |
| 124-NAMIBIA_S31_L001 | 124 | 404877 | 6155406 | 55.28 | 145651 |
