# Supplementary material for: Phenotypic and genomic characterization of antimicrobial resistance in Escherichia coli and Klebsiella spp. from livestock in Namibia
Source: PLoS One. 2026 Oct 13;21(10):e0359106. doi: 10.1371/journal.pone.0359106 (PMC13660907; doi:10.1371/journal.pone.0359106)
Supplement: Supplementary file 5 [file pone.0359106.s005.docx]

**Supplementary Table 3.** CARD output indicating some of the intrinsic chromosomal genes detected which contribute to resistance using multiple pathways.

| **Filename** | **Intrinsic genes** |
| --- | --- |
| 10-Ecoli_S19_L001 | *acrS, acrE, acrF, mphB, acrD, kdpE, bacA, tolC, Escherichia_coli_acrA,*  *acrB, cpxA, Escherichia_coli_ampH, pmrF, mdtP, mdtO, mdtN, emrB, emrA, emrR, Escherichia_coli_ampC1_ β-lactamase, Escherichia_coli_mdfA, emrY, emrK, evgA, evgS, marA, msbA, eptA, Escherichia_coli_ampC, H-NS, mdtH, mdtG, mdtA, mdtB, mdtC, baeS, baeR, yojI, Escherichia_coli_emrE, gadX, gadW, mdtF, mdtE, CRP, tet(A), dfrA5, sul1, mdtM* |
| 105-Ecoli_S4_L001 | *gadX, gadW, mdtF, mdtE, CRP, mdtM, Escherichia_coli_emrE, eptA,*  *mdtN, mdtO, mdtP, baeR, baeS, mdtC, mdtB, mdtA, ugd, kdpE, mphB, acrD, Escherichia_coli_ampC1_ β-lactamase, evgS, evgA, emrK, emrY, pmrF, yojI, Escherichia_coli_ampC, cpxA, emrB, emrA, emrR, H-NS, mdtH, mdtG, marA, tolC, bacA, acrS, acrE, acrF, msbA, Escherichia_coli_mdfA, Escherichia_coli_acrA, acrB, Escherichia_coli_ampH* |
| 106-Ecoli_S3_L001 | *TEM-141, Escherichia_coli_ampH, acrB, Escherichia_coli_acrA, kdpE,*  *mdtM, mdtE, mdtF, gadW, gadX, cpxA, mdtG, mdtH, emrB, emrA, emrR, CRP, pmrF, yojI, baeR, baeS, mdtC, mdtB, mdtA, H-NS, tolC, bacA, acrS, acrE, acrF, dfrA7, sul1, Escherichia_coli_mdfA, msbA, sul2, APH(3'')-Ib, APH(6)-Id, tet(A), mphB, acrD, Escherichia_coli_ampC1_ β-lactamase, evgS, evgA, emrK, emrY, CTX-M-55, APH(3')-Ia, marA, Escherichia_coli_ampC, eptA, mdtN, mdtO, mdtP* |

| 109-Ecoli_S5_L001 | *CRP, bacA, tolC, Escherichia_coli_ampC, eptA, mdtN, mdtO, mdtP, emrB, emrA, emrR, mdtG, mdtH, kdpE, mdtM, Escherichia_coli_mdfA, msbA, mdtE, mdtF, gadW, gadX, ugd, marA, H-NS, cpxA, mphB, mdtA, mdtB, mdtC, baeS, baeR, acrD, Escherichia_coli_ampC1_β-lactamase, evgS, evgA, emrK, emrY, yojI, pmrF, acrS, acrE, acrF, Escherichia_coli_acrA, acrB, Escherichia_coli_ampH* |
| --- | --- |
| 112-Ecoli_S6_L001 | *acrF, acrE, acrS, bacA, tolC, mdtE, mdtF, gadW, gadX, cpxA, CRP, ugd,*  *mdtA, mdtB, mdtC, baeS, baeR, yojI, pmrF, emrY, emrK, evgA, evgS, Escherichia_coli_ampC1_β-lactamase, acrD, mphB, Escherichia_coli_ampC, mdtM, H-NS, mdtH, mdtG, emrB, emrA, emrR, Escherichia_coli_acrA, acrB, Escherichia_coli_ampH, marA, msbA, Escherichia_coli_mdfA, mdtP, mdtO, mdtN, eptA, kdpE* |
| 113-Ecoli_S2_L001 | *msbA, kdpE, mdtM, Escherichia_coli_mdfA, mdtH, mdtG, acrF, acrE, acrS, bacA, tolC, emrB, emrA, emrR, marA, cpxA, mphB, acrD, Escherichia_coli_ampC1_β-lactamase, evgS, evgA, emrK, emrY, pmrF, yojI, baeR, baeS, mdtC, mdtB, mdtA, ugd, Escherichia_coli_ampC, eptA, mdtN, mdtO, mdtP, Escherichia_coli_ampH, acrB, Escherichia_coli_acrA, H-NS, CRP, mdtE, mdtF, gadW, gadX* |
| 39-Ecoli_S17_L001 | *emrB, emrA, emrR, baeR, baeS, mdtC, mdtB, mdtA, Escherichia_coli_ampH, marA, eptA, mdtE, mdtF, gadW, gadX, Escherichia_coli_ampC, yojI, kdpE, mdtN, mdtO, mdtP, acrS, acrE, acrF, tolC, bacA, Escherichia_coli_emrE, cpxA, pmrF, acrB, Escherichia_coli_acrA, Escherichia_coli_mdfA, mdtM, H-NS, emrY, emrK,* |

|  | *evgA, evgS, CRP, Escherichia_coli_ampC1_β-lactamase, acrD, mphB, msbA, mdtH, mdtG* |
| --- | --- |
| 55-Ecoli_S13_L001 | *CTX-M-14, Escherichia_coli_ampH, cpxA, pmrF, tolC, acrD, H-NS, evgA, evgS, Escherichia_coli_acrA, mdtN, mdtO, mdtB, eptA, mdtH, Escherichia_coli_emrE, baeR, baeS, acrS, acrE, mdtG, Escherichia_coli_ampC1_β-lactamase, bacA, CRP, kdpE, msbA, emrR, emrA, mdtM, mphB, mdtE, mdtF, gadW, gadX* |
| 75-Ecoli_S15_L001 | *emrB, emrA, emrR, cpxA, baeR, baeS, mdtC, mdtB, mdtA, ugd, H-NS, mdtH, mdtG, Escherichia_coli_mdfA, kdpE, yojI, pmrF, emrY, emrK, evgA, evgS, Escherichia_coli_ampC1_β-lactamase, acrD, mphB, tolC, bacA, acrS, acrE, acrF, marA, mdtP, mdtO, mdtN, eptA, Escherichia_coli_ampC,*  *mdtM, Escherichia_coli_acrA, acrB, Escherichia_coli_ampH, gadX, gadW,*  *mdtF, mdtE, CRP, msbA* |
| 78-Ecoli_S16_L001 | *kdpE, CRP, mdtG, mdtH, mdtM, acrF, acrE, acrS, marA, Escherichia_coli_mdfA, bacA, tolC, msbA, baeR, baeS, mdtC, mdtB, mdtA, emrY, emrK, evgA, evgS, mphB, acrD, Escherichia_coli_ampC1_β- lactamase, H-NS, Escherichia_coli_ampH, mdtP, mdtO, mdtN, eptA, emrR, emrA, emrB, gadX, gadW, mdtF, mdtE, yojI, cpxA, ugd, acrB, Escherichia_coli_acrA, Escherichia_coli_ampC* |
| 91-Ecoli_S14_L001 | *Escherichia_coli_ampC1_β-lactamase, acrD, mphB, Escherichia_coli_acrA, acrB, Escherichia_coli_ampH, kdpE, evgS, evgA, emrK, emrY, pmrF, yojI, baeR, baeS, mdtC, mdtB, mdtA, ugd, cpxA, Escherichia_coli_emrE, mdtM, Escherichia_coli_ampC, eptA, mdtN, mdtO,* |

*mdtP, H-NS, mdtH, mdtG, marA, tolC, bacA, acrS, acrE, acrF, msbA,*
