# Supplementary material for: Phenotypic and genomic characterization of antimicrobial resistance in Escherichia coli and Klebsiella spp. from livestock in Namibia
Source: PLoS One. 2026 Oct 13;21(10):e0359106. doi: 10.1371/journal.pone.0359106 (PMC13660907; doi:10.1371/journal.pone.0359106)
Supplement: Supplementary file 6 [file pone.0359106.s006.docx]

**Supplementary Table 4.** Virulence genes derived from *E. coli* isolates using the VFDB database.

| **Filename** | **Virulence genes** |
| --- | --- |
| 10-Ecoli_S19_L001 | *entA, entB, entE, entC, fepB, entS, fepD, fepG, fepC, entF, fes, fepA, entD, fdeC, ompA, ykgK/ecpR, yagZ/ecpA, yagY/ecpB, yagX/ecpC, yagW/ecpD, yagV/ecpE, espX4, espX5, gspC, gspD, gspE, gspF, gspG, gspH, gspI, gspJ, gspK, gspL, gspM, csgB, csgD, csgF, csgG, espR1, papX, espX1, iucA, iucB, iucC, iucD, iutA, iroN, iroE, iroD, iroC, iroB, espL1, fimH, fimG, fimF, fimD, fimC, fimI, fimA, fimE, fimB* |
| 105-Ecoli_S4_L001 | *fimB, fimE, fimA, fimI, fimC, fimD, fimF, fimG, fimH, espX5, espX4, entD, fepA, fes, entF, fepC, fepG, fepD, entS, fepB, entC, entE, entB, entA, espR1, gspC, gspD, gspE, gspF, gspG, gspH, gspI, gspJ, gspK, gspL, gspM, csgB, csgD, csgF, csgG, espL1, ompA, fdeC, ykgK/ecpR, yagZ/ecpA, yagY/ecpB, yagX/ecpC, yagW/ecpD, yagV/ecpE, espX1* |
| 106-Ecoli_S3_L001 | *entD, fepA, fes, entF, fepC, fepG, fepD, entS, fepB, entC, entE, entB, entA,*  *espL1, fimH, fimG, fimF, fimD, fimC, fimI, fimA, fimE, fimB, yagV/ecpE, yagW/ecpD, yagX/ecpC, yagY/ecpB, yagZ/ecpA, ykgK/ecpR, fdeC, csgG, csgF, csgD, csgB, gspL, gspM, espX5, espX4, espR1, hlyC, hlyA, hlyB, hlyD, faeE, faeD, ompA, espX1, astA* |
| 109-Ecoli_S5_L001 | *gspC, gspD, gspE, gspF, gspG, gspH, gspI, gspJ, gspK, gspL, gspM, csgG, csgF,*  *csgD, csgB, entD, fepA, fes, entF, fepC, fepG, fepD, entS, fepB, entC, entE, entB, entA, ompA, espX5, espX4, espR1, fdeC, espX1, espL1, espR4* |
| 112-Ecoli_S6_L001 | *espR1, fimB, fimE, fimA, fimI, fimC, fimD, fimF, fimG, fimH, espX1, csgB, csgD,*  *csgF, csgG, gspC, gspD, gspE, gspF, gspG, gspH, gspI, gspJ, gspK, gspL, gspM,* |

|  | *fdeC, ykgK/ecpR, yagZ/ecpA, yagY/ecpB, yagX/ecpC, yagW/ecpD, yagV/ecpE,*  *espL1, ompA, espX4, espX5, entA, entB, entE, entC, fepB, entS, fepD, fepG, fepC, entF, fes, fepA, entD* |
| --- | --- |
| 113-Ecoli_S2_L001 | *ompA, entA, entB, entE, entC, fepB, entS, fepD, fepG, fepC, entF, fes, fepA,*  *entD, fimH, fimG, fimF, fimD, fimC, fimI, fimA, fimE, fimB, csgB, csgD, csgF, csgG, gspC, gspD, gspE, gspF, gspG, gspH, gspI, gspJ, gspK, gspL, gspM, espR1, espL1, espX5, espX4, yagV/ecpE, yagW/ecpD, yagX/ecpC, yagY/ecpB, yagZ/ecpA, ykgK/ecpR, fdeC, espX1* |
| 39-Ecoli_S17_L001 | *ompA, cdtC, cdtB, cdtA, stxA, stx1B, entD, fepA, fes, entF, fepC, fepG, fepD, entS, fepB, entC, entE, entB, entA, espX5, espX4, espX1, hlyD, hlyB, hlyA, hlyC, gspM, gspL, gspK, gspJ, gspI, gspH, gspG, gspF, gspE, gspD, gspC, fimB, fimE, fimA, fimI, fimC, fimD, fimF, fimG, fimH, espR1, yagV/ecpE, yagW/ecpD, yagX/ecpC, yagY/ecpB, yagZ/ecpA, ykgK/ecpR, fdeC, espL1, csgB, csgD, csgF, csgG* |
| 55-Ecoli_S13_L001 | *yagW/ecpD, espL1, fimB, fimE, fimA, fimI, entA, entB, entE, espX5, fepD, fepG, fepC, entF, fes, yagV/ecpE, ykgK/ecpR, yagZ/ecpA, yagY/ecpB, csgF, csgD, csgB, entD, gspC, espX4, gspD, gspE, gspF, gspG, gspH, gspI, gspJ, gspK, fimD, ompA, entS, fepB, fimG, fimH* |
| 75-Ecoli_S15_L001 | *espY4, entA, entB, entE, entC, fepB, entS, fepD, fepG, fepC, entF, fes, fepA,*  *entD, aslA, fdeC, ykgK/ecpR, yagZ/ecpA, yagY/ecpB, yagX/ecpC, yagW/ecpD, yagV/ecpE, csgB, csgD, csgF, csgG, espX2, espR1, gspM, gspL, gspK, gspJ, gspI, gspH, gspG, gspF, gspE, gspD, gspC, espR4, espL1, espL4, espX4, espX5, fimB, fimE, fimA, fimI, fimC, fimD, fimF, fimG, fimH, espX1, espY2, espY3, chuV, chuU, shuY, shuX, chuW, shuT, shuA, shuS, ompA* |

| 78-Ecoli_S16_L001 | *espX1, espG, csgG, csgF, csgD, csgB, fimB, fimE, fimA, fimI, fimC, fimD, fimF,*  *fimG, fimH, espL1, ompA, yagV/ecpE, yagW/ecpD, yagX/ecpC, yagY/ecpB, yagZ/ecpA, ykgK/ecpR, fdeC, nleA, cif, nleH1, nleB2, espJ, entD, fepA, fes, entF, fepC, fepG, fepD, entS, fepB, entC, entE, entB, entA, espF, escG, escF, cesD2, espD, espA, sepL, escD, eae, cesT, map, cesF, espH, sepQ /escQ, escP, escO, escN, escV, cesL, escI, escJ, sepD, escC, cesD, etgA, escU, escT, escS, escR, escL, cesAB, escE, espX4, espX5, espR1, nleH2, nleF, gspC, gspD, gspE, gspF, gspG, gspH, gspI, gspJ, gspK, gspL, gspM, faeD, espM2, espW* |
| --- | --- |
| 91-Ecoli_S14_L001 | *entD, fepA, fes, entF, fepC, fepG, fepD, entS, fepB, entC, entE, entB, entA,*  *fdeC, ykgK/ecpR, yagZ/ecpA, yagY/ecpB, yagX/ecpC, yagW/ecpD, yagV/ecpE, espX1, espR1, fimH, fimG, fimF, fimD, fimC, fimI, fimA, fimE, fimB, espX5, espX4, csgB, csgD, csgF, csgG, espL1, gspM, gspL, gspK, gspJ, gspI, gspH, gspG, gspF, gspE, gspD, gspC, ompA* |
